# Comparison of Lysophospholipids and Bile Acids on the Growth Performance, Lipid Deposition, and Intestinal Health of Largemouth Bass (*Micropterus salmoides*)

**DOI:** 10.1155/2024/1518809

**Published:** 2024-02-07

**Authors:** Ming-Yang Bao, Zhe Wang, Waldo G. Nuez-Ortín, Guiping Zhao, Marleen Dehasque, Zhen-Yu Du, Mei-Ling Zhang

**Affiliations:** ^1^School of Life Sciences, East China Normal University, Shanghai 200241, China; ^2^Adisseo SAS, 10 Pl, du Général de Gaulle, Antony 92160, France; ^3^Adisseo Life Science (Shanghai) Co., Ltd., Shanghai 200241, China

## Abstract

Lysophospholipids (LPLs) and bile acids (BA) are commonly used as emulsifiers in aquaculture. This study investigated the effects of dietary supplementation of LPLs or BA on the growth performance, lipid deposition, and intestinal health of largemouth juveniles. Fish were randomly allotted into three groups in quadruplicate and fed with a basal diet (CON) or diets containing 300 mg/kg LPLs (LPLs), or 300 mg/kg commercially available BA product (BA) for 8 weeks. The results showed that compared with the control group, LPLs and BA supplemented groups showed a higher weight gain trend, and LPLs supplementation promoted the protein deposition in fish body. Both BA and LPLs supplementations helped to maintain liver health by decreasing the activities of aspartate aminotransferase and alanine aminotransferase in serum. Besides, LPLs supplementation decreased overall lipid deposition in terms of mesenteric fat index and liver lipid content. Furthermore, LPLs supplementation showed unique advantage in improving intestinal barrier, as characterized by the increased villus length and higher expression of the tight junction protein *zo-1* expression. LPLs supplementation also increased the alpha diversity index and the abundances of Proteobacteria in the intestinal microbiota which is positively correlated with the abundance of SCFA in the gut. These findings will promote the application of novel feed additives and especially provide a basis for the rational selection of emulsifiers in the aquaculture industry.

## 1. Introduction

With the rapid expansion of intensive aquaculture, the imbalance between the demand and supply of aquatic feeds has increased. Nonprotein energy ingredients, including carbohydrates and lipids, are commonly used in fish diets to spare protein [1]. Due to the limited utilization efficiency of carbohydrates in fish, lipids are more likely to be incorporated in aquatic feeds as the high-energy ingredients, especially for carnivorous fish [2]. However, it has been reported that high levels of dietary lipids also cause adverse effects on fish, including poor growth performance, extra lipid accumulation in the liver, and lower stress resistance [3]. Thus, it is important to increase the lipid utilization efficiency and decrease the negative effects induced by high-lipid diets.

The main steps for lipids absorption, transportation, and digestion in fish are similar to those in mammals [4]. Dietary lipids are hydrolyzed to free fatty acids and 2-monoacylglycerol in the gut by pancreatic lipase and then emulsified with bile acids (BA) before being absorbed in the enterocytes [4]. BA synthesized by the liver are essential for lipid metabolism. In aquaculture, BA salts have been used as emulsifiers [5]. Many studies indicated that supplementation with BA in the diet could reduce the overaccumulation of lipid in different fishes. It has been reported that dietary BA at the supplementation level of 200 mg/kg tended to decrease the lipid contents in both body and liver of tiger puffer (*Takifugu rubripes*) [6]. Likewise, appropriate BA supplementation level improved the digestion and absorption of lipids in juvenile large yellow croaker (*Larimichthys crocea*) [7] and juvenile rainbow trout (*Oncorhynchus mykiss*) [8]. Furthermore, 300 mg/kg BA supplementation could improve lipid utilization, antioxidant capacity, and intestinal health of largemouth bass fed a high-fat diet [9].

Lysophospholipids (LPLs) or lysolecithin are formed by the hydrolysis of lecithin by phospholipase which eliminates one molecule of fatty acid from the phospholipid molecules. LPLs are more hydrophilic than phospholipids, thus they are considered as an excellent emulsifier [10]. As animal feed additives, LPLs are able to emulsify lipids and promote the formation of small microclusters of lipids in the intestine, thus increasing the utilization efficiency of dietary lipids [10, 11]. Besides, LPLs can increase the fluidity and permeability of cell membrane, thus facilitating the absorption of other small-molecule nutrients [12]. In the study of channel catfish (*Ictalurus punctatus*), 250 mg/kg of dietary lysolecithin increased the antioxidative capacity and decreased liver lipid deposition [13]. In addition, 400 mg/kg dietary LPLs supplementation could promote the growth performance, improve hepatic lipid metabolism, and alleviate inflammation response in juvenile large yellow croaker (*Larimichthys crocea*) [14]. A previous study found that supplementation with 1 g/kg LPLs in low-protein (crude protein: 52.46%, crude lipid: 11.36%) or low-lipid (crude protein, 54.43%; crude lipid, 10.19%) diets could increase the activity of intestinal digestive enzymes, enhance the hepatic lipid metabolism, promote protein deposition, and modulate the intestinal flora of largemouth bass [15].

Largemouth bass (*Micropterus salmoides*) is an economically valuable carnivorous fish, referred to China Fisheries Statistical Yearbook. Its production in 2021 exceeded 700,000 tons in China. Researchers have assessed dietary LPLs and BA in largemouth bass and obtained favorable results, as mentioned above. However, no studies have been performed to compare the effects of LPLs and BA for fish species. Accordingly, the purpose of this study was to explore and compare the effects of dietary LPLs and BA at a commercially relevant inclusion level (300 mg/kg) on the growth performance, lipid deposition, and gut health of largemouth bass.

## 2. Materials and Methods

The experiment was carried out under the Guide for the Care and Use of Laboratory Animals in China. This research was approved by the Committee on the Ethics of Animal Experiments of East China Normal University (ECNU) (No. F20201002).

### 2.1. Experimental Design

M. salmonids juveniles were purchased from Sanshui Platinum Aquafarm (Foshan, Guangdong, China) and acclimatized for 14 days before the experiment. Fish (initial weight 21.1 ± 1.2 g) were randomly divided into three dietary treatments (four tanks per group, 20 fish per tank), including control diet (CON group), CON diet supplemented with 300 mg/kg BA (BA group), and CON diet supplemented with 300 mg/kg LPLs (LPLs group) (Nutri-lyso, Adisseo). The ingredients and proximate composition of the diets are presented in Table 1. All ingredients were ground and sieved through a 425 *μ*m mesh, mixed with water before pelletization using a twin-screwed extruder. The diameter of the extruded pellets was made in 3 and 5 mm. Finally, the floating pellets were sprayed with oil, air-dried, and stored at −20°C until use. The fish were fed manually to satiety twice a day (8:00 and 17:00) for 8 weeks. Fish were counted and weighted fortnightly. Feed intake was recorded to calculate the feed conversion ratio (FCR). Feces were siphoned out from the bottom of the tanks before feeding, and one-third of the water in the tank was renewed daily. During the feeding trial, water quality parameters including temperature (27 ± 1°C), pH (7.4 ± 0.19), dissolved oxygen (6.0 ± 1.2 mg/L), and ammonia nitrogen (<0.02 mg/L) were routinely measured (recorded weekly).

### 2.2. Growth Performance

At the end of the feeding trial, fish were starved for 12 hr and euthanized with MS-222 (200 mg/L; Sigma, USA). The fish number in each tank was recorded to calculate the survival rate. The weight of fish body, feed intake, visceral mass, mesenteric fat, and liver were measured to calculate the weight gain (WG), FCR, condition factor (CF), viscerosomatic index (VSI), mesenteric fat index (MFI), hepatopancreas somatic indices (HSI), and protein deposition rate (PDR) according to the following formulae:

(1) $$\begin{array}{c} \text{Survival rate }\left(\%\right)=\frac{\text{Final fish number}}{\text{Initial fish number}}\times 100, \end{array}$$

(2) $$\begin{array}{c} \text{WG }\left(\%\right)=\frac{\left[\text{Final body weight (g)}-\text{Initial body weight (g)}\right]}{\text{Initial body weight (g) }}\times 100, \end{array}$$

(3) $$\begin{array}{c} \text{FCR}=\frac{\text{Feed intake (g)}}{\left[\text{Final body weight (g)}-\text{Initial body weight (g)}\right]}, \end{array}$$

(4) $$\begin{array}{c} \text{CF }\left(\text{g}/{\text{cm}}^{3}\right)=\frac{\text{Final body weight (g)}}{\text{Final body length }{\left(\text{cm}\right)}^{3}}\times 100, \end{array}$$

(5) $$\begin{array}{c} \text{VSI }\left(\%\right)=\frac{\text{Viscera weight (g)}}{\text{Final body weight (g)}}\times 100, \end{array}$$

(6) $$\begin{array}{c} \text{MFI }\left(\%\right)=\frac{\text{Mesenteric fat weight (g)}}{\text{Final body weight (g)}}\times 100, \end{array}$$

(7) $$\begin{array}{c} \text{HSI }\left(\%\right)=\frac{\text{Liver weight (g)}}{\text{Final body weight (g)}}\times 100, \end{array}$$

(8) $$\begin{array}{c} \text{PDR }\left(\%\right)=\frac{\left[\text{Final body weight (g)}-\text{Initial body weight (g)}\right]\times \text{Final protein content of fish }\left(\%\right)}{\left[\text{Feed intake (g)}\times \text{Protein content in feed }\left(\%\right)\right]}\times 100. \end{array}$$

### 2.3. Proximate Analysis

Six fish from each treatment were picked for body composition analyses according to the standard methods [16]. Briefly, moisture content was analyzed by oven drying to a constant weight at 105°C for 12 hr; protein content was determined by a semiautomatic Kjeldahl System (FOSS, Sweden); lipids were extracted using chloroform/methanol (2 : 1, v/v). The lipid composition in the liver was detected by thin-layer chromatography (TLC) [17, 18]. In detail, the total lipids were extracted, dissolved in 10 *μ*L methylene chloride, and dripped on the activated silica gel thin layer (F254, Merck, German). Phospholipids (phosphatidylethanolamine (PE), phosphatidylinositol (PI), phosphatidylserine (PS), and phosphatidylcholine (PC)) were separated in a solvent system consisting of methyl acetate/isopropyl acid chloroform/methanol/0.25% KCl (25/25/25/10/9, v/v/v/v). Neutral lipids (TG, diacylglycerol (DG), and monoglyceride (MG)) and phospholipids were separated in a solvent system consisting of heptane/ether/acetic acid (55/45/1, v/v/v). The silica gel thin-layer plates were put into an oven at 50°C for 30 min and then dyed with iodine for 20 min. The lipid compositions were scanned and quantified by KH-3000 TLC Scanner (Kezhe, Shanghai, China).

### 2.4. Serum Biochemical and Digestive Enzyme Analyses

Blood samples were collected from the caudal vein of four fish per treatment, centrifuged (2,500x *g*, 10 min) to separate serum, and then stored at −80°C until analysis. The separated serum was used to detect the activity of aspartate aminotransferase (AST; Cat. No. C010-2) and alanine aminotransferase (ALT; Cat. No. C009-2) according to the kit instructions (Jiancheng Biotech. Co., Nanjing, China).

The intestinal samples were collected from six fish per treatment and homogenized with 1x PBS to obtain a 1/10 (w/v) intestine homogenate, centrifuged at 4°C (3,500x *g*, 10 min) and then stored at −80°C before analysis. The digestive enzymes activity 1,4-*α*-D-Glucan-glucanohydrolase (*α*-amylase; Cat. No. C016-1) and lipase (LPS, Cat. No. A054-2) of intestine homogenate were measured using commercial kits following the manufacturer's instructions (Jiancheng Biotech. Co., Nanjing, China).

### 2.5. Histological Analysis

The foregut tissues from three fish per treatment were immediately fixed in 4% paraformaldehyde and then embedded in paraffin. Five micrometers sections were stained with hematoxylin and eosin and then washed with 70% alcohol. The stained samples were observed under a light microscope (Nikon Ds Ri2, Tokyo Metropolis, Japan). The length and width of intestinal villi were measured by the image software (Nis-Elements F package version 4.60).

### 2.6. Intestinal Microbiota Analysis

Intestinal contents from three fish per treatment were collected and pooled for total DNA extraction by using the Soil DNA Kit (D5625, Omega, Norcross, GA, USA) according to the manufacturer's instructions. The V3–V4 regions of the 16S rRNA gene were amplified by PCR using the primer 338F (5′-ACTCCTACGGGAGGCAGCA-3′) and the primer 806R (5′-GGACTACHVGGGTWTCTAAT-3′). PCR products were sequenced on Illumina MiSeq/NovaSeq (Shanghai Personal Biotechnology Co., Ltd., China). The Quantitative Insights Into Microbial Ecology (QIIME, v2) pipeline was used for sequence quality filtering, denoising, and chimera checking. The nonchimera sequences were reclustered at 97% to generate OTU table. Alpha diversity indices including Chao1, Simpson, observed species, and Shannon index were calculated by QIIME. Principal component analysis and heat-map analysis were performed using R software.

### 2.7. Short-Chain Fatty Acids (SCFAs) Detection

The gut contents (200 mg) of three fish per treatment were suspended with 0.5 mL of distilled water. Fifty milliliters 50% sulfuric was then added to the mixture for acidification for 30 s. The acidified supernatant was supplemented with 0.3 mL diethyl ether to extract SCFAs. After centrifugation at 12,000x *g* for 10 min, the supernatants were collected and analyzed in a gas chromatograph (GC7900, TianMei Scientific Instrument, China) under the following conditions: An initial column temperature of 100°C, held for 2 min, increased at a rate of 5°C/min to 180°C, and then held for 2 min; the flow rate was kept at 1 mL/min; the inlet temperature was set to 220°C; and the sample amount was 1 *μ*L with nitrogen as the carrier gas [19]. The contents of acetic acid, propionic acid, and butyric acid were measured according to their external standard curve.

### 2.8. Quantitative Real-Time PCR Analysis

Six fish from each group were sampled for gene expression analysis. The total RNA of the samples was isolated by Tri Pure Reagent (Aidlab, Beijing, China). The quality and quantity of RNA were tested by Nanodrop 2000 Spectrophotometer (Thermo, Waltham, USA). RNA with 260/280 nm absorbance ratios between 1.8 and 2.0 was used. cDNA was synthesized using a PrimeScript™ RT Reagent Kit (Takara) in an S1000TM Thermal Cycler (Bio-Rad). The PCR amplification program was the following: 95°C for 10 min, 40 cycles of 95°C for 5 s and 60°C for 15 s. Primer specificity was analyzed by melting curve analysis. The primer sequences are shown in Table 2. Elongation factor 1*α (ef1α*) and *β-actin* were used as the reference genes. The relative mRNA expressions were calculated by the 2^−*ΔΔ*CT^ method [20].

### 2.9. Statistical Analysis

Statistical analysis of all data was performed using SPSS 23.0. Statistical comparisons between groups were conducted by the one-way analysis of variance (ANOVA). The data were represented as mean ± SEM (standard error of the mean). Differences were considered significant at *P* < 0.05.

## 3. Results

### 3.1. Growth Performance

**Figure 1:**
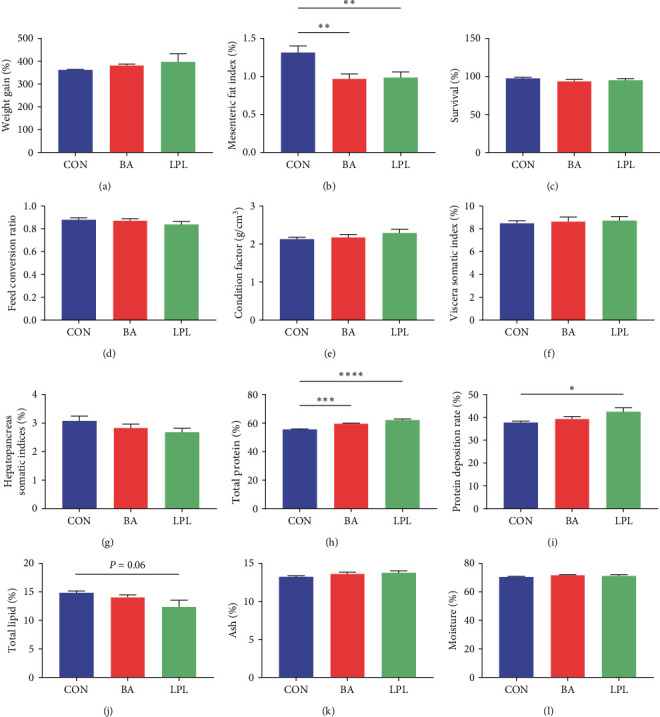
The effects of LPLs and BA on growth performance of *M. salmoides*. (a) Weight gain, (b) mesenteric fat index, (c) survival rate, (d) feed conversion ratio, (e) condition factor, (f) viscera somatic index, (g) hepatopancreas somatic indices, (h) total protein, (i) protein deposition rate, (j) total lipid, (k) ash, and (l) moisture. The data are presented as mean ± SEM (*n* = 4–9) ( ^*∗*^*P* < 0.05;  ^*∗∗*^*P* < 0.01;  ^*∗∗∗*^*P* < 0.001;  ^*∗∗∗∗*^*P* < 0.0001). WG, weight gain; MFI, mesenteric fat index; FCR, feed conversion ratio; CF, condition factor; VSI, viscera somatic index; HSI, hepatopancreas somatic indices.

At the end of the 8-week feeding trial, the WG in LPLs group showed a trend to increase by 11% compared with the control, but it was not statistically significant (Figure 1(a)). Dietary BA supplementation trend to increase the WG by 5% compared with the control (Figure 1(a)). Both BA and LPLs supplementation significantly reduced the MFI (*P* < 0.05) (Figure 1(b)), whereas no significant difference was observed in the survival rate, FCR, CF, VSI, and HSI (*P* < 0.05) (Figure 1(c)–1(g)). For whole-body composition, the proportion of total protein significantly increased in the LPLs and BA group compared with the control (*P* < 0.05) (Figure 1(h)), and the protein deposition rate significantly increased in LPLs group compared with the control (*P* < 0.05) (Figure 1(i)). The proportion of total lipid showed a decreasing tendency in the LPLs group (*P*=0.06) (Figure 1(j)). The contents of moisture and ash were similar among all three groups (*P* > 0.05) (Figures 1(k) and 1(l)).

### 3.2. Effects of LPLs and BA on Liver Health

**Figure 2:**
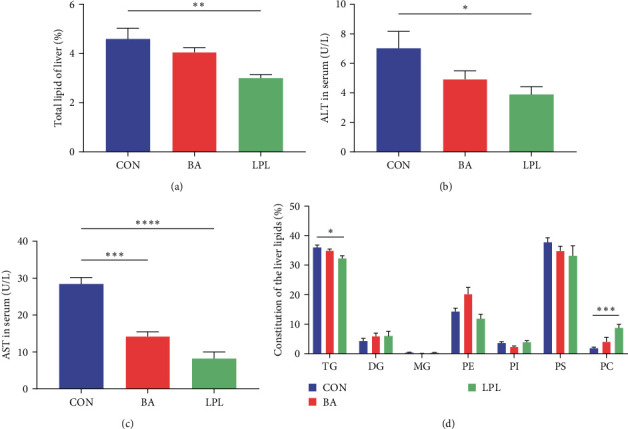
The effects of LPLs and BA on liver health of *M. salmoides*: (a) total lipid of liver, (b) alanine aminotransferase in serum, (c) aspartate aminotransferase in serum, and (d) constitution of liver lipid detected by thin-layer chromatography (TLC) analysis. The data are presented as mean ± SEM (*n* = 4–6) ( ^*∗*^*P* < 0.05;  ^*∗∗*^*P* < 0.01;  ^*∗∗∗*^*P* < 0.001;  ^*∗∗∗∗*^*P* < 0.0001). ALT, alanine aminotransferase; AST, aspartate aminotransferase; TG, triglycerides; DG, diacylglycerol; MG, monoglyceride; PE, phosphatidylethanolamine; PI, phosphatidylinositol; PS, phosphatidylserine; PC, phosphatidylcholin.

Dietary LPLs supplementation significantly decreased the total lipid in the liver compared with the control (*P* < 0.05) (Figure 2(a)). The concentrations of AST and ALT in serum significantly decreased in the LPLs group compared with the control (*P* < 0.05) (Figures 2(b) and 2(c)). Supplementation with BA could significantly decrease the concentration of AST in serum (*P* < 0.05) (Figure 2(b)).

TLC was performed to analyze the composition of lipids in the liver. Compared with the control, LPLs supplementation significantly decreased the TG content but increased the PC content in the liver (*P* < 0.05) (Figure 2(d)). These results indicated that LPLs application decreased lipid deposition and changed the lipid composition in the liver.

### 3.3. Effects of LPLs and BA on Gut Health

**Figure 3:**
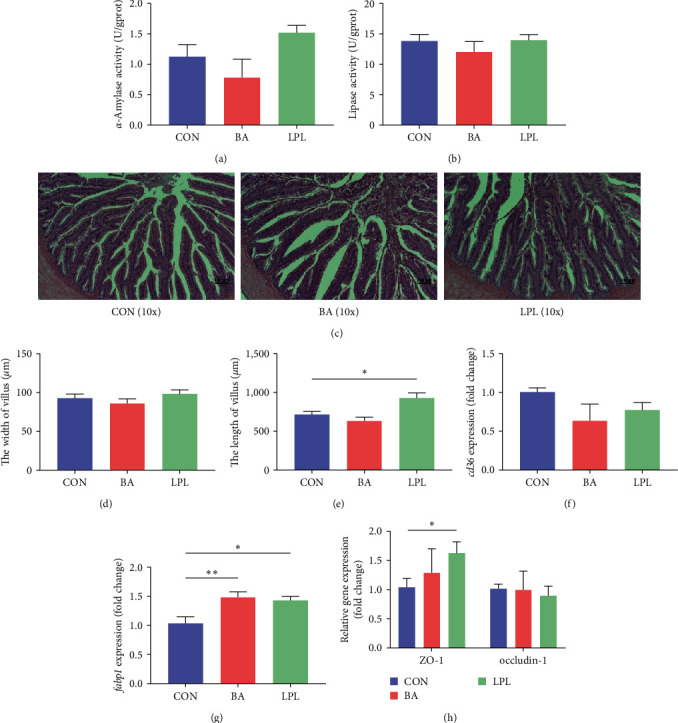
The effects of BA and LPLs on gut health of *M. salmoides*: (a) *α*-amylase activity, (b) lipase activity, (c) H&E sections, (d) the width of villus, (e) the length of villus, (f) the expression level of *cd36*, (g) the expression level of *fabp1*, and (h) the expression level of zo-1 and occluding-1. The data are presented as mean ± SEM (*n* = 4–6) ( ^*∗*^*P* < 0.05;  ^*∗∗*^*P* < 0.01).

The LPLs or BA did not influence the activities of lipase and *α*-amylase in the intestine (*P* > 0.05) (Figures 3(a) and 3(b)). Hematoxylin and eosin staining of intestine tissue showed that the villus width was similar among groups (*P* > 0.05) (Figures 3(c) and 3(d)), but LPLs supplementation significantly increased the villus length compared with the control (*P* < 0.05) (Figure 3(e)).

The effects of BA and LPLs on the expression of lipid transport-related genes, *cd36* (thrombospondin receptor) and *fabp1* (fatty acid-binding protein 1), were detected. LPLs and BA significantly increased *fabp1* expression but did not affect *cd36* expression in the intestine (*P* < 0.05) (Figure 3(g)). LPLs supplementation significantly increased *zo-1* expression in the intestine (*P* < 0.05) (Figure 3(h)), though the *occluding-1* expression is similar among groups (*P* > 0.05) (Figure 3(h)). These results suggested that LPLs could improve the intestinal morphology and barrier functions as well as the absorption efficiency of fatty acids.

### 3.4. Effects of LPLs and BA on Microbial Composition and SCFAs Concentration in the Intestine

**Figure 4:**
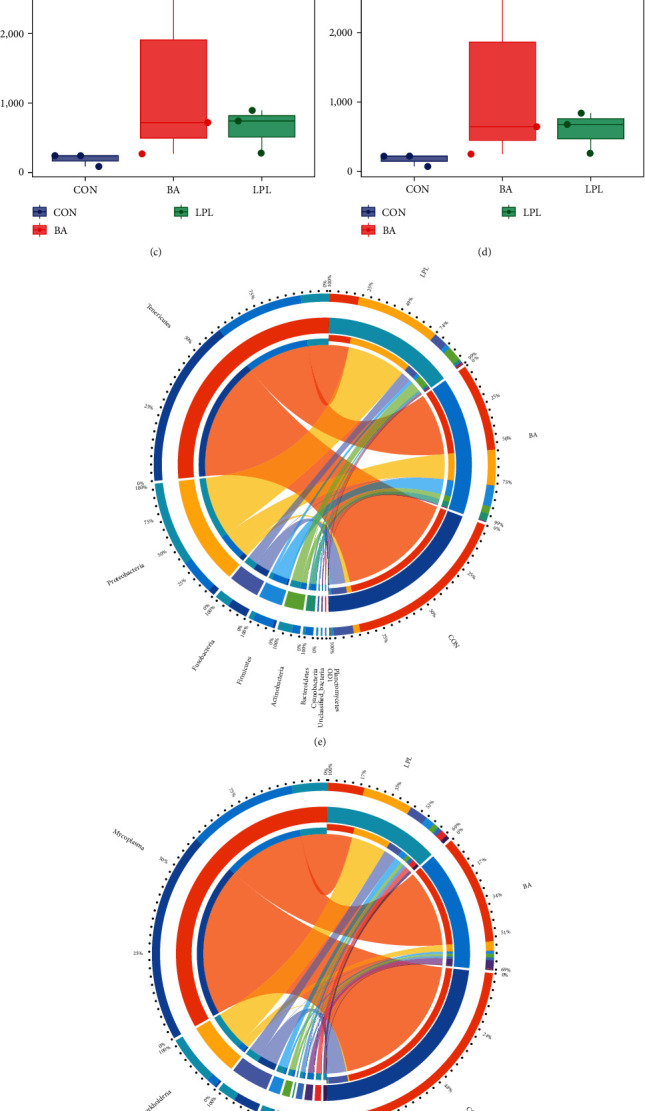
The effects of BA and LPLs on microbial composition and SCFAs concentration in the intestine of *M. salmoides*: (a) Simpson index, (b) Shannon index, (c) Chao1 index, (d) observed species, (e) the composition of gut microbiota at the phylum level, (f) the composition of gut microbiota at the genus level, (g) the top 10 microbial species in abundance at the genus level, and (h) the concentration of acetic acid in intestinal contents. The data are presented as mean ± SEM (*n* = 3) ( ^*∗*^*P* < 0.05;  ^*∗∗*^*P* < 0.01;  ^*∗∗∗*^*P* < 0.001).

The microbiota composition of each group was determined by sequencing the 16S rRNA gene. Dietary LPLs supplementation significantly increased the Simpson and Shannon indices compared with the control group (*P* < 0.05) (Figures 4(a) and 4(b)), whereas Chao1 and observed species indices were similar among groups (*P* > 0.05) (Figures 4(c) and 4(d)).

The composition of intestinal microbial species was analyzed. At the phylum level, Tenericutes was the most dominant bacterial phylum in the control and BA groups, whereas the most abundant phylum was Proteobacteria in the LPLs group, followed by Tenericutes (Figure 4(e)). At the genus level, *Mycoplasma* was dominant in CON and BA group. The most abundant taxa was *Burkholderia* in LPLs group. At the genus level, dietary LPLs supplementation significantly increased the abundances of *Burkholderia, Ochrobactrum, Halomonas*, and *Acinetobacter* while the abundance of *Mycoplasma* decreased compared with the control, dietary BA supplementation significantly increased the level of *Halomonas* compared with the control (*P* < 0.05) (Figures 4(f) and 4(g)).

The concentration of SCFAs in intestinal contents was detected by GC-MS. Compared with the control, the concentration of acetic acid was significantly increased in LPLs and BA groups (*P* < 0.05), but the contents of propionic acid and butyric acid were below the detection limit in all these groups (Figure 4(h)).

## 4. Discussion

BA and LPLs are widely used as emulsifiers in aquafeed industry, but the comparison between these two emulsifiers has not been conducted. The present study compared the beneficial effects of dietary LPLs and BA on the growth performance, lipid deposition, and gut health of largemouth bass at the level of 300 mg/kg, a commercially relevant inclusion. The results showed that dietary BA and LPLs had the potential to increase the weight gain of largemouth bass. The growth promotion effect of dietary BA was also observed in Nile tilapia (*Oreochromis niloticus*) and yellow croaker (*Larimichthys crocea*), which may be mediated partly by the emulsification of lipids [7, 21, 22]. Similarly, a study showed that dietary LPLs increased the growth performance of juvenile turbot (*Scophthalmus maximus L*.) partially by increasing the lipid utilization efficiency [10]. A recent study employed proteomics to identify the effects of dietary LPLs on Atlantic salmon (*Salmo salar*), and it was found that the energy obtained via the Krebs cycle of the liver was increased in the LPLs addition group, explaining the possible reason for the growth-promoting effect of LPLs [23].

As emulsifiers, it is unsurprising that both BA and LPLs decreased the MFI of largemouth bass compared with the control group. Studies have found that dietary BA or LPLs supplementation could decrease the lipid deposition in different fishes. The possible reasons are (1) improving the emulsification of lipids and (2) accelerating the digestion and absorption efficiency of the intestine [15, 23–28]. Thus, we detected the expression levels of lipid transport-related genes in the intestine. We found that dietary BA and LPLs upregulated the expression level of lipid transport gene *fabp1* in the intestine of fish. Fabp1 plays a vital role in fatty acid uptake and transport, suggesting that dietary LPLs and BA could improve the absorption efficiency of lipids in the intestine of largemouth bass [29]. Further, evidence is provided that digestion and absorption of nutrients depend on the activity of the digestive enzymes [30]. The activity of intestinal lipase and *α*-amylase were detected, but they were not affected by the addition of LPLs or BA. It has been found that the emulsifiers could improve the emulsification of lipids which may benefit the absorption of fatty acids [31], which may be the reason for the increased expression of *fabp1*. Taken together, these results indicated that dietary LPLs and BA could increase the fatty acids capacity but decrease the lipid deposition of largemouth bass.

Notably, body composition analyses showed that dietary LPLs increased the content of total protein and the protein deposition rate of fish compared with the control, while dietary BA did not. Similar effects of LPLs were observed in juvenile amberjack (*seriola dumerili*), yellow croaker (*Larmichthys crocea*), and blunt snout bream (*Megalobrama amblycephala*) [32–34]. Of note, we found LPLs supplementation increased the PC content in the liver lipid. A study in Nile tilapia (*Oreochromis niloticus*) showed that 400 mg/kg PC supplementation increased the total serum protein levels [35]. These changes may be related to the increase of total protein of fish. Meanwhile, dietary LPLs decreased the concentrations of AST and ALT in serum compared with the control. It has been reported that high levels of AST and ALT may cause physiological or pathological conditions such as liver damage and an increase of protein catabolism [36]. We have detected the expression level of mTOR (data not shown), but no significant difference was found. Thus, more investigations are needed to explore the possible mechanisms.

Considering that the epithelium of the intestinal mucosa acts as a physical barrier and is critical for the proper function of the intestine [37]. The influence of dietary BA and LPLs on gut health was compared. In the current study, the addition of LPLs significantly increased the length of intestinal villus and increased the expression level of intestinal tight junction protein *zo-1* of largemouth bass. Similarly, the addition of LPLs at the level of 2 g/kg increased the gut villus height of rainbow trout [38]. It has been found that dietary supplementation of 1 g/kg LPLs could maintain the intestinal barrier function by improving vesicle trafficking in Atlantic salmon (*Salmo salar*), thus promoting nutrient absorption and utilization [23]. These results suggested that supplementation with LPLs improved intestinal integrity and barrier function, which help to maintain gut health.

The commensal intestinal microbiota of fish is associated with host growth, development, and metabolism [39]. Results in the previous study showed that dietary emulsifiers can influence the host–microbiota relationship resulting in the changes of host gut microbial composition [40]. In our study, dietary LPLs and BA supplementations modulated the gut microbiota compositions of largemouth bass. Specifically, LPLs treatment increased the Simpson and Shannon indices of gut microbiota, indicating an increase in species diversity. A previous study showed that LPLs supplementation in feed trend to increase the alpha diversity index of the intestinal microbiota in largemouth bass larvae [41]. This is similar to our findings. Generally, the loss of gut microbial diversity is implicated in the dysregulation of intestinal homeostasis [42]. At the phylum level, the dominant microbes were Tenericutes, Proteobacteria, Fusobacteria, and Firmicutes in each group. Proteobacteria, Firmicutes, and Fusobacteria are widespread in the intestine of fish [15]. Specifically, dietary LPLs supplementation trend to reduce the relative abundance of Tenericutes and increase the abundance of Proteobacteria. Effects of dietary LPLs supplementation in low-lipid feeds on largemouth bass were consistent with our findings [15]. At the genus level, the relative abundance of *Burkholderia* is highest in the LPLs group, a potentially beneficial bacterium [43]. Dietary LPLs supplementation also inhibited the growth of the potential pathogen *Mycoplasma* which dominates in the CON group [44]. The relative abundance of *Ochrobactrum, Halomonas*, and *Acinetobacter* were also increased in the LPLs group at the genus level. In conclusion, LPLs treatment contributed to the maintenance of a healthy and diversified microbiota composition of largemouth bass.

Gut microbiota could produce beneficial metabolites such as SCFAs by fermenting carbohydrates from the diets [45]. Studies have shown that SCFAs participate in numerous physiological processes of the host, including the intestinal barrier development, nutrient metabolism, energy expenditure, and immune responses [46]. In the current study, both LPLs and BA supplementations significantly increased the content of acetic acid, which could improve gut inflammatory responses and metabolism homeostasis in fishes [19, 47, 48]. This may be associated with an increase in the abundances of Proteobacteria. The content of acetic acid is positively correlated with the abundance of Proteobacteria in intestine [49]. It was therefore reasonable to suspect that the remodeling gut microbiota and related metabolites accounted for the beneficial effect of LPLs and BA observed in this study, though further investigation is needed.

## 5. Conclusion

The current study compared the influence of two emulsifiers—LPLs and BA—on largemouth bass. LPLs and BA supplementations trend to increase the weight gain. LPLs supplementation also promoted the protein deposition in fish body. Both BA and LPLs supplementations helped to maintain liver health and decreased lipid deposition in terms of mesenteric fat index. Furthermore, BA and LPLs supplementations altered the microbial composition and increased acetic acid contents in the gut and LPLs showed a unique advantage in improving the intestinal barrier.

## Figures and Tables

**Table 1:** Ingredients and proximate composition of the experimental diets of largemouth bass (dry matter).

Ingredients (g/kg)	CON	BA	LPLs
Fish meal^a^	450	450	450
Chicken meat powder^b^	50	50	50
Soybean meal^c^	95	95	95
Flour^d^	100	100	100
Wheat gluten meal^e^	50	50	50
Tapioca starch^f^	30	30	30
Soybean oil^g^	60	60	60
Fish dissolved pulp^h^	50	50	50
Spray-dried blood cells^i^	30	30	30
Monocalcium phosphate^j^	15	15	15
Premix^k^	10	10	10
Cellulose	59.7	59.4	59.4
Y_2_O_3_	0.3	0.3	0.3
Lysophospholipid^l^	—	—	0.3
Bile acids^m^	—	0.3	—
Ash	11.31	11.26	11.26
Crude protein	51.9	52.8	52.0
Crude lipids	14.0	14.0	13.3

^a^Peruvian fishmeal (65% protein), main antinutrients (trypsin inhibitor, cysteine protease inhibitor), produced by HaiXing ZhengFa Feed Sales Co., Ltd., Hebei, China. ^b^Chicken meat powder (55% protein), main antinutrients (cysteine protease inhibitor), produced by Dongchen Biotechnology Co., Ltd., Shandong, China. ^c^Soybean meal (46% protein), main antinutrients (trypsin inhibitor, phytic acid, and hemagglutinin), produced by Chinatex Grains and OILS Rizhao Co., Ltd., Shandong, China. ^d^Produced by Yihai Kerry Arawana Holdings Co., Ltd., Shanghai, China. ^e^Produced by Tezhen trading Co., Ltd., Zhengzhou, China. ^f^Produced by Ganzhiyuan sugar Co., Ltd., Nanjing, China. ^g^Produced by Yihai Kerry Arawana Holdings Co., Ltd., Shanghai, China. ^h^Produced by Xingyan New Material Technology Co., Ltd., Hubei, China. ^i^Produced by Yurun Biotechnology (Donghai) Co., Ltd., Jiangsu, China. ^j^Produced by Wanbang Chemical Technology Co., Ltd. Henan, China. ^k^Premix composition: (1) one kilogram of vitamin premixes contained: Vitamin A 500,000 IU, Vitamin D3 50,000 IU, Vitamin E 2,500 mg, Vitamin K3 1,000 mg, Vitamin B1 5,000 mg, Vitamin B2 5,000 mg, Vitamin B6 5,000 mg, Vitamin B12 5 mg, inositol 25,000 mg, pantothenic acid 10,000 mg, choline 100,000 mg, Niacin 25,000 mg, folic acid 1,000 mg, biotin 250 mg, Vitamin C 10,000 mg; (2) one kilogram of mineral premixes contained: CaCO_3_ 314.0 g, KH_2_PO_4_ 469.3 g, MgSO_4_ · 7H_2_O 147.4 g, NaCl 49.8 g, Fe (II) gluconate 10.9 g, MnSO_4_ · H_2_O 3.12 g, ZnSO_4_ · 7H_2_O 4.67 g, CuSO_4_ · 5H_2_O 0.62 g, KI 0.16 g, CoCl_2_ · 6H_2_O 0.08 g, NH_4_ molybdate 0.06 g, NaSeO_3_ 0.02 g, the ratio of vitamin premix to mineral premix is 1 : 1. ^l^Provided by Adisseo Life Science Co., Ltd., (Shanghai, China). ^m^Extracted from porcine bile; Provided by Adisseo Life Science Co., Ltd., Shanghai, China. Purity 99%; containing 69.9% hyodeoxycholic acid, 18.9% chenodeoxycholic acid, and 7.8% hyocholic acid.

**Table 2:** Primer sequences of the genes used for qRT-PCR.

Gene name	Primer sequence (5′-3′)	Gene registration number
*ef1α*	F: TGCTGCTGGTGTTGGTGAGTT	XM_038724778.1
	R: TTCTGGCTGTAAGGGGGCTC	

*β-actin*	F: AAAGGGAAATCGTGCGTGAC	XM_038695351.1
	R: AAGGAAGGCTGGAAGAGGG	

*fabp 1*	F: GAACCTCAAGGAGAGCCAGAA	XM_038725022.1
	R: CACCGTCCACCGAGATAATAGT	

*zo-1*	F: CAACAAAGACTGCCTTCCCAC	XM_038700548.1
	R: CTGTAGCACCTGTAAGCCCAT	

*occludin-1*	F: TAATGTCCTCCAGGCCCAATG	XM_038734216.1
	R: CGCAAGCAAATATACCCACGG	

*cd36*	F: ACACAAGAGGGAAGAGGCGT	XM_038739147.1
	R: ACTGGCTTTGCGCCATACT	

*ef1α*, elongation factor 1-alpha-like; *β-actin*, actin beta 2; *fabp1*, fatty acid binding protein 1; *zo-1*, tight junction protein 1a; occludin-1, occludin a; *cd36*, thrombospondin receptor.

## Data Availability

The data that support this finding will be available upon reasonable request from the corresponding author.
